# Detection of immunoglobulin (Ig) A antibodies against porcine epidemic diarrhea virus (PEDV) in fecal and serum samples

**DOI:** 10.1016/j.mex.2015.10.001

**Published:** 2015-10-13

**Authors:** Priscilla F. Gerber, Tanja Opriessnig

**Affiliations:** a The Roslin Institute and The Royal (Dick) School of Veterinary Studies, University of Edinburgh, Midlothian, UK; b Department of Veterinary Diagnostic and Production Animal Medicine, Iowa State University, Ames, IA, USA

**Keywords:** Porcine epidemic diarrhea virus, IgA, Diagnostic, Serology, ELISA, Feces, Serum

## Abstract

Many assays for detection of antibodies against porcine epidemic diarrhea virus (PEDV) are based on detection of neutralizing antibodies or immunoglobulin (Ig) G in serum samples. However, due to the particular features of the mucosal immune system, presence of serum antibodies against enteric pathogens, such as PEDV, not always correlates with protection. In contrast, anti-PEDV IgA antibodies correlate with protection against subsequent challenges. An indirect PEDV IgA ELISA was previously developed to monitor IgA levels in colostrum and milk samples. In the present paper we describe an adaptation of the protocol for detection of IgA antibodies in serum and fecal samples.

• The adapted protocol will aid in future assessment of protective levels of humoral response against PEDV infection by measuring IgA levels in serum and fecal samples.
• Fecal samples are non-invasive and easy to collect at any time by animal caretakers and therefore offering advantages over the serum sample collection procedure.
• A strong positive correlation between the anti-PEDV levels in fecal and serum samples was identified; however, detection of IgA antibodies was often more successful in serum than in paired fecal samples due to overall lower sample-to-positive (S/P) ratios for the latter sample type.

## Graphical abstract

**Figure:**
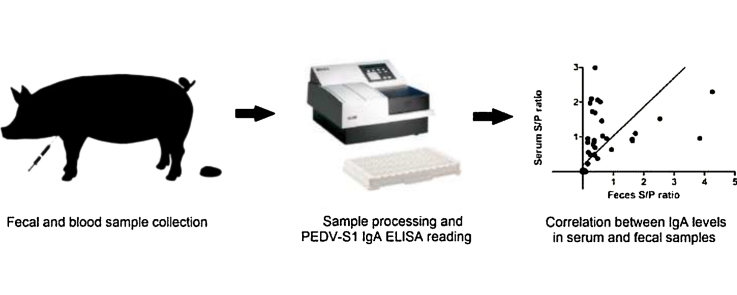

## Method details

The present study was designed to adapt an indirect ELISA protocol for the detection of anti-porcine epidemic diarrhea virus (PEDV) IgA in serum and fecal samples to compare the capability of detection of IgA in the two different matrix types. The spike gene S1 domain (aa 1–781) of a genogroup 2 prototype U.S. PEDV expressed in a mammalian vector was used as antigen [1].

### Control samples

Fecal and serum controls for the IgA PEDV S1-ELISA were obtained from a pig infected experimentally with PEDV (positive control) or from a pig sham-infected (negative control) at 28 days post infection or sham infection (dpi) [2], [3]. The experimental protocol was approved by the Iowa State University Institutional Animal Care and Use Committee (Approval No. 2-14-7742-S). In brief, 3-week-old PEDV negative pigs were inoculated with 10 ml of the 5th passage of PEDV isolate 13-19338E at a tissue culture infective dose (TCID_50_) of 5 × 10^2^ per ml via the oral route or sham-inoculated with 10 ml of virus negative culture medium via the oral route. Rectal swabs were collected at 0, 1, 3 and 7 dpi. Serum samples were collected at 0, 7, 14, 21 and 28 dpi, fecal samples were collected at 28 dpi, and the samples were aliquoted in 1.5 ml microcentrifuge tubes and stored at −80 °C until use. The pigs were humanely euthanized and necropsied at 28 dpi. Presence or absence of PEDV infection was confirmed by RT-PCR on rectal swabs [2], [3] and by an IgG immunofluorescence assay (IFA) in serum samples as routinely performed at the Iowa State University Veterinary Diagnostic Lab (ISU-VDL).

### Buffers

*Coating buffer*. 50 mM carbonate buffer, pH 9.6
*Blocking buffer.* Phosphate buffered saline (PBS) pH 7.2 containing 0.05% Tween 20 (PBST), 1% bovine serum albumin, 10% sucrose (Gibco, Life Technologies)
*Assay diluent.* PBST 10% goat serum (Gibco, Life Technologies)
*Washing buffer*. PBST

### Plate coating and blocking

1. Microtiter plates (Maxisorp, Nunc) were coated with 0.44 ng per well (100 μl) of the S1 antigen diluted in coating buffer and incubated overnight at 4 °C.
2. After three washes with washing buffer, the plates were incubated with blocking buffer for 2 h at 22 °C. Plates were used immediately after blocking or dried at 37 °C for 2–4 h and stored at 4 °C until use.

### Preparation of samples

*Fecal samples*. Fresh fecal samples and controls were diluted at 1:10 w/v (0.1 g in 0.9 ml) with assay diluent, thoroughly homogenized by vortexing for 30 s and centrifuged at 4000 × *g* for 10 min.
*Serum samples*. Serum samples and controls were diluted at 1:100 (3 μl in 300 μl) with assay diluent. Samples were mixed thoroughly before being distributed on the plates.

### ELISA procedure

#### Primary antibody incubation

*Fecal samples*. Clarified diluted fecal samples or controls were added to each well and incubated at 4 °C for 16–18 h. After incubation, plates were washed 3 times with washing buffer.
*Serum samples*. Diluted samples or controls (100 μl) were added to each well and incubated at 37 °C for 1 h. Controls were tested in duplicate. After incubation, plates were washed 3 times with washing buffer.

#### Secondary antibody incubation

a. Anti-porcine IgA antibodies (Goat anti-swine IgA HRP-conjugated, Bethyl) were diluted at 1:5000 in assay diluent.
b. The secondary antibody (100 μl) was added to each well and incubate at 37 °C for 30 min.
c. After incubation, plates were washed 3 times with washing buffer.

#### Third incubation using substrate

a. TMB substrate (100 μl; SureBlue™ TMB Microwell Peroxidase Substrate Kit, KPL Inc.) was added to each well and incubated at 22 °C for 20 min in the dark.
b. The reaction was stopped by addition of 50 μl of 2 M sulphuric acid solution per well.
c. Absorbance of the samples and controls was read at 450 nm (ELx808 Absorbance Microplate Reader; BioTek).

#### Test interpretation

A run on a particular plate was considered to be valid when the negative control optical density (OD) was lower than 0.15 and the positive control OD value was higher than 0.60. The S/P ratio was calculated using the following formula: S/P ratio = [(Sample OD − Negative sample OD)/(Positive sample OD − Negative sample OD)].

For the IgA ELISA on fecal samples an S/P ratio less than 0.13 was considered negative and an S/P ratio higher or equal to 0.13 was considered positive. For serum samples an S/P ratio less than 0.14 was considered negative and an S/P ratio higher or equal to 0.14 was considered positive.

## Method validation

For the optimization of the assay, a total of 69 paired serum and fecal samples from pigs with known PEDV exposure [2], [3] were used. Source of the pigs and inoculation were similar to what has been described under “control samples”. Specifically, samples collected at 28 dpi from 31 PEDV infected pigs and 38 sham-inoculated negative control pigs were utilized [2], [3]. Samples were tested in single wells in at least two independent runs and the mean S/P value was used for each sample.

**Fig. 1:**
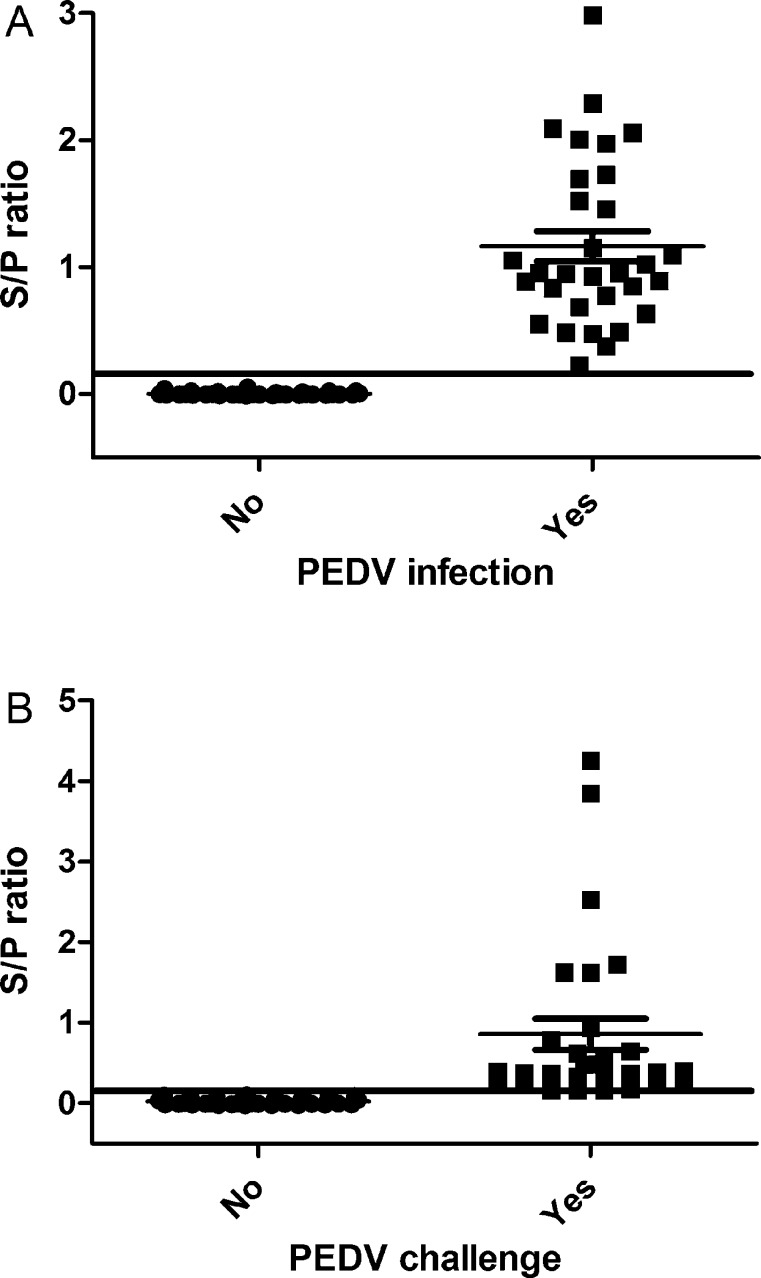
Distribution of anti-IgA porcine epidemic diarrhea virus (PEDV) ELISA sample to positive (S/P) ratios on (A) serum and (B) fecal samples distributed according to PEDV challenge status of the animal (no, yes) relative to the assay cut off (full line). An S/P ratio higher or equal to 0.14 was considered positive for serum samples and an S/P ratio higher or equal to 0.13 was considered positive for fecal samples.

The cut off value for the IgA ELISA was calculated by the mean S/P ratios of PEDV negative samples plus three standard deviations (SD). The cut off points were determined to be a sample S/P ratio of 0.13 (average S/P ± SD, 0.02 ± 0.03) for the fecal ELISA and sample S/P ratio of 0.14 (average S/P ± SD, 0.00 ± 0.01) for the serum ELISA. These values were further evaluated using a receiver operator characteristic (ROC) analysis (Fig. 1). The analysis was performed using GraphPad Prism v. 6.01 (GraphPad Software, La Jolla, CA, USA).

**Fig. 2:**
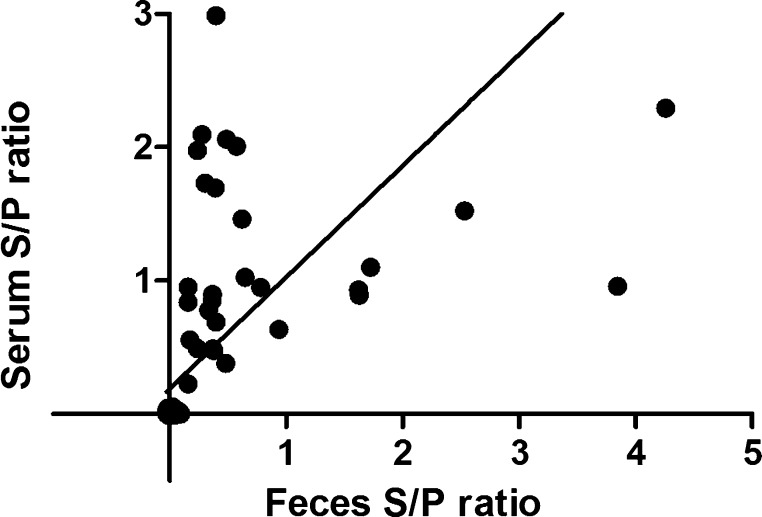
Correlation of anti-IgA porcine epidemic diarrhea virus (PEDV) sample to positive (S/P) ratios on paired serum and fecal samples.

A correlation between S/P values in serum and the corresponding fecal sample from a given pig was determined using Spearman's rank correlation method. A significant positive correlation was found between fecal and serum anti-IgA levels (*r* = 0.7889; *p* < 0.001, Fig. 2).

## Background

PEDV, a member of the genus *Alphacoronavirus* in the family *Coronaviridae*, causes acute diarrhea, vomiting, and dehydration in pigs of any age with often high mortality in neonatal piglets. PEDV strains can be divided into genogroup 1 and genogroup 2 based on significant amino acid differences in the N-terminal domain of the S gene [4]. PEDV infection has resulted in high economic losses in Asian pig industries, the disease has been reported in 2013 in North America and in 2014 in South America [5] and recent outbreaks have been described in Europe during 2015. Most PEDV strains circulating in Europe and in Asia prior to 2010 belong to genogroup 1 while the more recent outbreaks were associated with PEDV genogroup 2. PEDV genogroup 2 can be divided in at least two major clusters, one containing the strains similar to the ones that emerged in the US in 2013 (U.S. PEDV prototype strain) and the other cluster containing PEDV strains with distinct insertions and deletions in the spike gene (S INDEL strains) [6]. Although the antigen used for the ELISA development in the present study was based on the U.S. PEDV prototype strain, the same protocol can be applied for S1 antigens based on different strain sequences that may be more relevant in a certain geographic location.

Due to the similarities in clinical signs with other enteric pathogens and as part of eradication attempts, accurate and timely diagnosis of PEDV infection is important. A number of commercial and *in-house* PEDV ELISA techniques have been developed for detection of antigen in feces or antibodies in serum and colostrum [1], [7], [8], [9], [10], [11], [12], [13], [14].

Due to the particular features of the mucosal immune system, the presence of serum antibodies against pathogens that replicate primarily in mucosal surfaces, such as PEDV and other coronaviruses, is not always correlated with protection; rather, detection of these antibodies only indicates that individuals had previous contact with infectious microorganisms [15], [16], [17] or were vaccinated. Therefore, for mucosal infections, measurement of localized IgA immune responses is critical to evaluate protection derived either by vaccines or by previous pathogen exposure. Indeed, a previous study has shown that anti-PEDV IgA antibodies in serum samples correlated with protection against a subsequent challenge 21 days after the first inoculation [18]. In veterinary diagnostics, serum is a standard sample for serological testing; however, in case of enteric pathogens, testing fecal samples perhaps would allow a better assessment of local immunity.

Fecal samples have many advantages over serum samples including ease of collection by animal caretakers, a non-invasive collection procedure, and collection of samples on an as-needed basis. Drawbacks include the presence of inhibitors and a lower concentration of analytes. A good correlation was found between the levels of PEDV IgA antibodies in serum and fecal samples indicating that either fecal material or serum could be used for assessing local PEDV immunity in pig herds. Future studies should also assess oral fluids for suitability as a sample type to assess herd immunity. However, similar to fecal samples, inhibitors and low analyte concentrations may impair detection of antibodies in this sample type [19]. In addition, pen-based oral fluid samples represent composite samples from 25 up to 350 pigs and establishing the rates of individual protection appears not feasible.
